# Cape Gooseberry (*Physalis peruviana* L.) Volatile Compounds Determination by Vacuum-Assisted Sorbent Extraction (VASE)—Selected Aspects

**DOI:** 10.3390/molecules29153477

**Published:** 2024-07-25

**Authors:** Henryk H. Jeleń, Monika Marcinkowska

**Affiliations:** Faculty of Food Science and Nutrition, Poznań University of Life Sciences, Wojska Polskiego 31, 60-624 Poznań, Poland

**Keywords:** vacuum, sorbent extraction, VASE, *Physalis peruviana* L., cape gooseberry, microextraction

## Abstract

Vacuum-Assisted Sorbent Extraction (VASE) is a novel extraction technique that uses vacuum to facilitate the transfer of volatile compounds from the matrix to the sorbent. This technique was explored for extraction of volatiles from cape gooseberry fruit, for both qualitative and quantitative analyses. Selected extraction parameters were tested: sample size, extraction temperature and time, influence of tissue disintegration on release of volatiles, and also addition of Ag^+1^ ions in the form of AgNO_3_ to stop enzymatic formation of volatile compounds. For selected conditions (10 g sample, extraction for 30 min. at 40 °C of volatiles from blended fruit) quantitative aspects were explored. Twenty-two compounds of cape gooseberry were tested. The method was characterized with a very good linearity in a range of 10–5000 µg/kg and good reproducibility. The experiments proved the usefulness of VASE in both volatile profiling and quantitative analyses of cape gooseberry and in prospective other fruit.

## 1. Introduction

VASE (Vacuum-Assisted Sorbent Extraction) is a relatively new extraction technology that has been developed in the last decade with a high potential for exploration in the field of food analysis. However, there are only few applications described in the literature focused on the analysis of volatile compounds of various foods [1,2,3]. VASE is based on using vacuum to transfer volatile and semi-volatile compounds into the sorbent material placed in a sorbent pen, a unique device that enables evacuation of air from an extraction vial. Sorbent-based techniques dominate the field of volatile extraction from various matrices, and approaches such as solid phase microextraction (SPME) in different variants (fibers, thin films, arrows), needle-based devices (needle trap devices (NTD), in-tube extraction (ITEX), and stir bars play a leading role in volatile compound extraction [4]. The use of vacuum in sorbent-based extraction techniques improves headspace extraction kinetics, from which mainly low-volatility organic compounds benefit. According to theory, the total amount of analyte extracted at equilibrium will be the same at ambient pressure and under vacuum. However, vacuum-assisted extraction influences most significantly the analytes with Henry constants (K_H_) below 1.6 × 10^−4^ atm m^3^ mol^−1^, where the mass transfer between the sample matrix and headspace is the rate-determining step [5,6].

Cape gooseberry (*Physalis peruviana* L.) is a fruit belonging to the *Solanaceae* family, genus *Physalis*, and originates from the Andean region of South America. It is cultivated mainly in Colombia and Peru, but also in South Africa, Kenya, Australia, New Zealand, Malaysia, and China [7,8]. The fruit of *P. peruviana* L. is usually consumed fresh, and the fruit can be also used in jams and jellies. It has a relatively long shelf life if stored properly; at 8 °C this can be as long as 62 days [9]. *P. peruviana* L. has been used in traditional Colombian medicine with attributed health activities, such as diuretic, antiseptic, sedative, and analgesic properties, and elimination of throat troubles or intestinal parasites [7]. Several review papers have been published on the bioactive phytochemicals in *P. peruviana* L. and its potential as a functional food [10,11]. Cape gooseberry contains high levels of vitamin C (up. to 929 mg 100 g^−1^), total phenolics (up to 935 mg 100 g^−1^, as gallic acid equivalent), and carotenoids (β-carotene, up to 1074.7 mg 100 g^−1^ FW), though the literature data exhibit a large spread. It is also a good source of vitamins B3 and B6, phytosterols, withanolides, physalins, and minerals [8,12,13]. Detailed analysis of non-volatiles (phenolics, flavonoids, sucrose esters of mainly isobutanol and dodecanol, and steroids), as well as terpenes of different volatility, was conducted using a metabolomic platform [14]. Using the same platform, withanolides have been the subject of interest [15]. The potential of anticancer and antioxidant properties of gooseberry has been investigated [16,17,18].

Volatile compounds of *Physalis peruviana* L. have been the subject of investigation aimed at the profiling of volatile compounds by different techniques, as well as the determination of the aroma active fraction of volatiles. Not only volatiles in free form, but also those bound as glycosides were investigated [19]. Berger at al. [20] used liquid/liquid extraction of homogenized fruit by pentane/dichloromethane (2/1 *v*/*v*) followed by fractionation of extracts on a SiO_2_ column, re-extraction of the organic phase with Na_2_CO_3_, followed by extraction with diethyl ether and C18 fractionation to determine glycosidally bound volatiles. Esters were the dominant fraction (53 compounds), followed by alcohol terpenes (28 compounds), acids (26), ketones (13), aldehydes (12), lactones (7), and hydrocarbon terpenes (5). Several (8–12) odor impressions were recognized and related to detected aroma compounds [20]. Liquid/liquid extraction was also used to isolate volatiles from blended cape gooseberry. Dichloromethane was used as a solvent and alcohols (21) were the dominant fraction (almost 4 mg/kg), followed by lactones (6 compounds, 2.1 mg/kg), esters (23 compounds, 1.1 mg/kg), and terpenes (11 compounds, 1.0 mg/kg). Lactones were the most odor-active compounds [21]. When liquid/liquid extraction (LLE) was compared to SPME of blended fruit (using DVB/CAR/PDMS fiber), alcohols were the dominant fraction (44% peak area for LLE and 39% for SPME), but for other groups of compounds there were significant differences noted resulting from the diverse character of extraction techniques: lactones (24%) in LLE were only 2% in SPME, acids were correspondingly 5% and 0.9%, aldehydes were 1.6% and 7.1%, and esters were 11.7% and 38.5% [22]. SPME was also used in the comparison of wild and cultivated fruits with notable differences in monoterpene esters, alcohols, and some hydrocarbons in favor of cultivated fruit [23]. In-tube extraction (ITEX) [24] was used for quantitation of 24 compounds extracted using this method from cape gooseberry [25], including mainly terpenes, but also aldehydes, alcohols, and esters. The method was linear in a range of 5(25)–500 µg/kg (R^2^ = 0.951–0.999) with LOD of 0.035–6.90 µg/kg depending on the compound [19]. When the sensomic approach was used to identify odor-active compounds in cape gooseberry after solvent-assisted flavor evaporation (SAFE), the compounds with the highest odor activity value (OAV) were ethyl butanoate, β-linalool, (E)-non-2-enal, (2E, 6Z)-nona-2,6-dienal, hexanal, ethyl octanoate, and furaneol (all with OAV > 50 [26].

To investigate further the possibilities of the extraction of volatile compounds from cape gooseberry, VASE was tested in this paper. The investigation involved extraction conditions, method development, examination of the influence of the fruit sample, pretreatment, quenching enzymatic reactions responsible for volatile compound formation, and finally testing of the quantitative aspects of VASE.

## 2. Results and Discussion

Sample size plays a crucial role in sorbent-based extraction techniques as it determines the amounts of analyte adsorbed on a sorbent, thus influencing the limits of detection. It can also result in sorbent overload with all the consequences related to chromatographic analysis. From small samples in relation to sorbent capacity, exhaustive extraction is possible. In sorbent-based methods, extraction temperature and time also influence the profile of extracted compounds. These factors influence sample throughput and also affect the quantitative aspects of the developed method. The final issue, often overlooked, is the sampling process from biological systems, which often after tissue disintegration enables enzymes to react with non-volatile precursors, leading to the release of volatiles. Therefore, the process is highly dynamic and, as a result, the profile of volatiles can be significantly different depending on the sample pretreatment used. Various extraction methods are used for profiling volatile compounds in non-target analyses, resulting in effects highly dependent on extraction methods. Moreover, quantitative aspects are to be considered if a particular technique should be used for target analysis of particular volatile compounds.

### 2.1. Influence of Sample Size on Volatile Compounds

**Figure 1:**
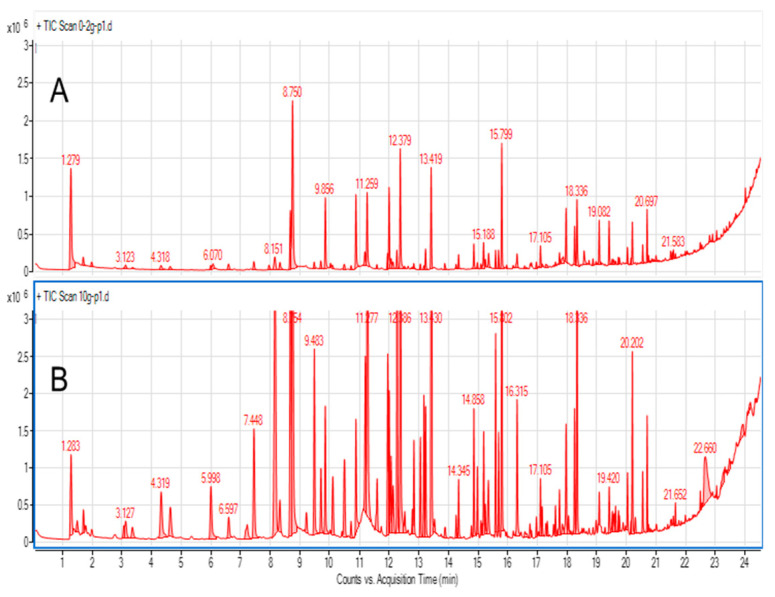
GC-MS chromatograms (total ion current (TIC)) of gooseberry obtained from 0.2 g (**A**) and 10.0 g (**B**) fruit samples.

**Figure 2:**
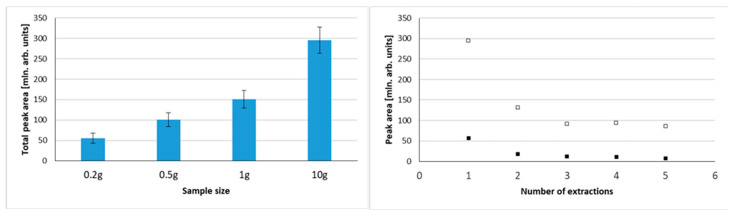
Influence of sample size on the total volatile compounds extracted (expressed as a total peak area) (**left** graph) and influence of number of extractions performed on a single sample on the total peak area of extracted volatiles for 10 g sample (▫) and 0.2 g sample (▪); (**right** graph).

To test the optimal sample size, samples of 0.2 g, 0.5 g, 1.0 g, and 10.0 g were tested, primarily to explore the influence of sample weight on total peak area. Figure 1 shows chromatograms obtained using 0.2 g and 10.0 g samples of blended cape gooseberry using 30 min. extraction at 40 °C. A matter that should be considered is the quantity of analytes (and potential interferences vs. sorbent amount/capacity. When total peak areas were compared for different sample sizes, a nonlinear increase was noted (Figure 2). For 0.5 g samples, the total area was roughly twice as big as for 0.2 g. However, the total peak area increased by one-third when a 1.0 g sample was compared to a 0.5 g sample, and for a 10.0 g sample the total peak area was twice as big as for 1.0 g. This illustrates that the sorption capacity of the sorbent is a limiting step in extraction efficiency in this experiment, especially in case of big samples. However, VASE differs from dynamic headspace/purge and trap sorption as there is no free flow of gas through the sorbent, so as a consequence volatiles are deposited in a front of the sorbent. This minimizes channeling effects, and facilitates better desorption without the need for refocusing with liquid nitrogen. One must be aware that a 10 g sample size is considerably high, as for many microextraction methods the sample size rarely exceeds 1 g, or even smaller samples of 50–200 µg are analyzed. From the point of view of sample uniformity in heterogenous biological material (as in fruit skin, flesh, seeds, etc.), such small samples should be avoided, unless liquid nitrogen milling is used to obtain a powdered uniform form. For small sample sizes, which are usually analyzed by SPME, the injection is performed in a split-less mode, guaranteeing the complete transfer of volatiles into a gas chromatograph. In the case of VASE, due to technical reasons, split injections are performed (in this research 1:25). Therefore, the system does not suffer from column overloading and symmetrical peak shapes are achieved (Figure 1).

Increasing the sample size allows the collection of higher numbers of volatile compounds and/or higher quantities to be extracted. Figure 2 shows the influence of sample size on total peak area, and also illustrates the possibility of multiple extractions from the same sample using VASE. The multiple extraction was tested for 0.2 g and 10.0 g samples, indicating the gradual decrease in peak areas in the first three extractions, and thus a substantial depletion of analytes from the sample, regardless of the sample size. The extractions were performed one after another (considering extraction time, analysis, and bakeout times) using the same sorbent pen. This indicates a high capacity of the VASE sorbent. On the other hand, it indicates that, regardless of the sample size (although especially visible in bigger samples), there is not enough sorbent to perform exhaustive extraction with a single sampling. Nonetheless, it also indicates the potential possibility of using VASE for multiple headspace extraction for quantitative analyses. This approach is used for static headspace analysis (MHE), as well as for solid phase microextraction (SPME) [27,28].

### 2.2. Influence of Extraction Temperature and Time on Volatile Compounds

**Figure 3:**
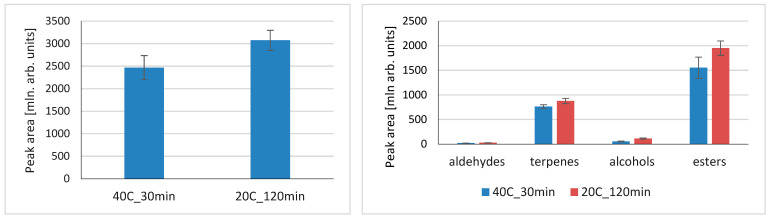
Influence of extraction time and temperature on total compounds’ peak areas and particular compound classes extracted using VASE.

To explore the different approaches in terms of extraction temperature/time in VASE, two radically different approaches were compared—40 °C extraction for 30 min. vs. extraction at room temperature (20 °C) for 120 min. The results are presented in Figure 3. When total peak areas were compared, longer extraction at room temperature resulted in higher values. For particular compound classes, longer extraction at room temperature favored more compounds being adsorbed. Extraction temperature and time are crucial parameters that influence the efficiency of extraction, and, as a result, the sensitivity of analysis. Heating the sample increases Henry’s volatility constant, K_H_, which results in higher headspace concentration and shorter equilibration times [5]. Higher temperatures enable better migration of volatiles from the matrix to headspace; however, this depends on the character of the matrix and compounds. As a result, extraction of polar compounds from a water matrix (high-K_c/w_ compounds) is improved by increased temperature. This is not the case for low-K_c/w_ compounds, where temperature increases have little influence on compounds with low water affinity. Considering the specificity of VASE as an extraction technique, vacuum needs to be taken into consideration; a high temperature decreases vacuum, and therefore the benefits of VASE in the extraction of compounds having a high Henry’s constant are theoretically weakened. In sorbent-based methods, where extraction is based on partition coefficients, the profiles of extracted compounds usually differ, being highly dependent on extraction time, as well as compound type and factors that affect extraction kinetics. As observed for SPME, a high time dependance of proportions of mono- to sesquiterpenes in black pepper was observed [29]. The results shown for the combination of temperature and time provide some hints for a strategy for volatile extraction, which can be tailored for specific compound groups, where particular parameters will provide higher method sensitivity. The other factor to be considered is the overall analysis time, and although the extractions for the sample sequence can overlap, assuming that the GC/MS run time is much shorter than the extraction time, higher throughputs may be favored with shortened extraction times. When the profiles of volatiles extracted in tested conditions were compared, there were no notable differences in the presence/absence of particular compounds, but their abundances varied. Therefore, the choice of particular extraction conditions provides similar qualitative results, but can be a subject of further studies in terms of tuning the method for particular compounds’ quantitative analyses. To perform a detailed optimization of the influence of time/temperature on the extracted compounds, a design of experiment methods could be applied. However, in the case of this study, a general picture of radically different approaches was presented, with the similarities/differences between them.

### 2.3. Influence of Tissue Treatment on Volatile Compounds

**Figure 4:**
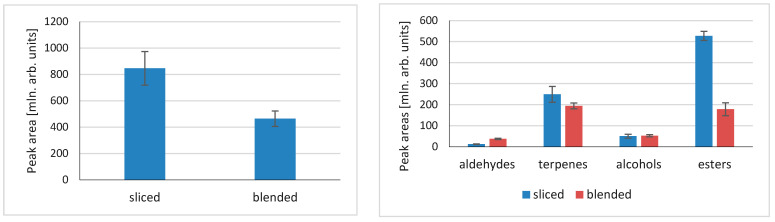
Influence of physical sample pretreatment (slicing vs. blending) on the volatile compounds released from gooseberry tissue.

Extraction of volatile compounds from fruit and vegetables usually involves a factor that is often overlooked while developing the extraction method. Volatile compounds in fruit and vegetables that are mainly generated based on the interaction of non-volatile precursors and enzymes that catalyze their formation are usually located in separate cellular sections/compartments. Additionally, the reactions take place in tissue disintegration, which is conducted by mechanical operations, such as cutting, chopping, or blending. Examples of such processes can be unsaturated fatty acid oxidation mediated by the LOX/HPL enzyme system, glucosinolate enzymatic degradation by myrosinase in Brassica vegetables, or alkyl(alkenyl)-cysteine sulfoxide degradation by alliinase in *Allium*. Figure 4 illustrates the differences in peak areas for gooseberry that were sliced or blended for both total peak areas and particular compound classes.

When slicing is compared to blending, there is a substantial difference in total compounds formed, with those for sliced samples being almost twice as high as those for blended. More specific information can be obtained when particular compound classes are compared (Figure 4). The quantities of compounds differ when various groups are compared. There is almost no difference in alcohols released, whereas for aldehydes there is a significant, trifold increase in aldehydes formed after blending. More specifically, the amount of hexanal after blending was 1.7 times higher than for slicing; for (E)-2-hexenal, the value was 7.6 times higher; for (Z)-3-hexene-1-ol, it was 3.9 times higher; and for 1-hexanol, it was 1.3 times higher. This confirms the crucial influence of blending, and thus the tissue disintegration method, on the activity of lipoxygenase (LOX). It also suggests that greater alcohol dehydrogenase, which catalyzes the oxidation of alcohols to corresponding aldehydes, is more active after tissue blending. Contrarily, blending decreases the quantity of esters released, and a similar result is observed for terpenes. However, in this case it might be related to physical changes in the tissue structure rather than enzymatic reactions.

**Figure 5:**
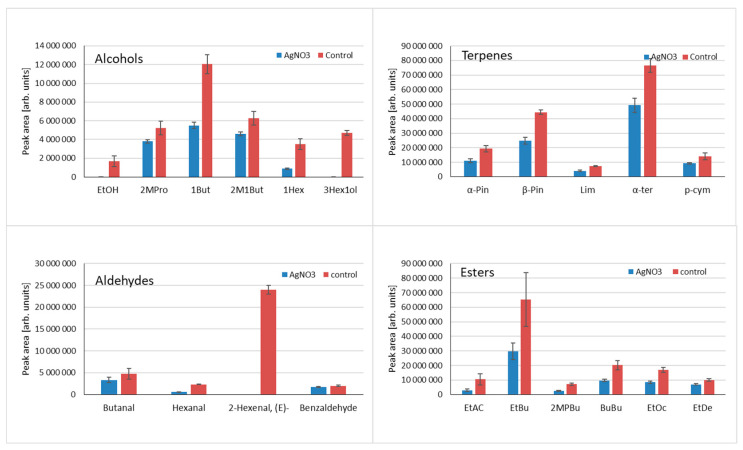
Influence of Ag^+1^ in the form of AgNO_3_ on the release of volatile compounds from blended gooseberry tissue for main classes of compounds: alcohols (ethanol, 2-methyl-1-propanol, 1-butanol, 2-methyl-1-butanol, 1-hexanol, 3-hexen-1-ol); terpenes (α-pinene, β-pinene, limonene, α-terpinolene, p-cymene); aldehydes (butanal, hexanal, (E)-2-hexenal, benzaldehyde); esters (ethyl acetate, ethyl butyrate, 2-methylpropyl butyrate, butyl butyrate, ethyl octanoate, ethyl decanoate).

The formation of volatile compounds in fruit and vegetables is often a combination of various enzyme-mediated pathways of different dynamics and the susceptibility to environmental factors. Therefore, depending on tissue treatment and methods used (if any) to quench enzymatic reactions, the profile of extracted volatile compounds can significantly differ. To overcome the problem of dynamic volatile compound formation as a result of tissue disintegration, various methods are used to quench enzymatic reactions; however, this practice is relatively rarely used. Usually, the addition of different salts is preferred, which influences enzyme activity; deprivation of water in the sample results from adding salts, and liquid nitrogen is used to stop the reaction. It has to be stressed that thermal inactivation of enzymes using heat is not preferred as it influences the profile of volatiles [30]. The addition of AgNO_3_, which was already tested for kohlrabi [31], was tested in this research to evaluate its influence on volatile compound formation as a result of enzymatic reactions. Data are shown in Figure 5. For all tested alcohols, the peak areas are higher for control samples where no AgNO_3_ was used. Differences vary depending on compounds: for ethanol and 3-hexene-1-ol, AgNO_3_ completely quenched the reaction—no compounds were detected. For the remaining compounds, higher amounts were observed for control samples. When aldehydes were examined in all samples, the addition of AgNO_3_ decreased the quantity of compounds. It can be concluded that AgNO_3_ influences the LOX pathway, as the quantities of LOX-derived compounds, such as (E)-2-hexenal, hexanal, and 3-hexen-1-ol, are significantly higher for control samples. In case of 3-hexene-1-ol and (E)-2-hexanal, complete inhibition of their formation by AgNO_3_ was noted. As can be seen for terpenes and esters, the addition of AgNO_3_ results in a significant decrease in emitted volatile compounds, which was noted for all the tested compounds. For terpenes, the suppression of their formation can be related to the release of these compounds from non-volatile precursors (glycosidally bound terpenes) by native glucosidase, which can be affected by AgNO_3_. The pool of sugar-bound volatiles in many fruits is a known phenomenon, and it frequently exceeds the pool of volatiles present in the free (unbound) form [32,33].

To conclude, the profile of the extract depends not only on the specificity and character of extraction parameters, but is also highly related to the tissue state and sample preparation, especially in terms of inactivation of enzymatic processes related to release of volatiles from non-volatile precursors.

However, a substantial question emerges: Is the profile of volatile compounds altered/quenched with the addition of some enzyme inhibitors, a natural aroma/volatile compound profile for a given fruit or vegetable? In fact, when eating fruit or vegetables, one faces and appreciates the dynamic formation of aroma, so it can be assumed that this is the natural profile of aroma/volatile compounds, and no quenching of enzymatic reactions is required as it can bias the “real” profile. On the other hand, especially in quantitative analyses, the ongoing reactions in the sample preparation process have to be taken into consideration. Due to enzymatic activity, the profile of volatiles in the samples that were kept in an autosampler during a long sequence could be substantially different, which was a result of various times when the fruit tissue was exposed to enzymatic activity.

Considering the conditions tested above for quantitative purposes, a sample size of 10 g was chosen, fruit samples were blended (which facilitated better distribution of the added standards in quantitation), and extraction at 40 °C for 30 min. was selected, improving the throughput in the analytical process.

### 2.4. Quantitative Aspects

**Figure 6:**
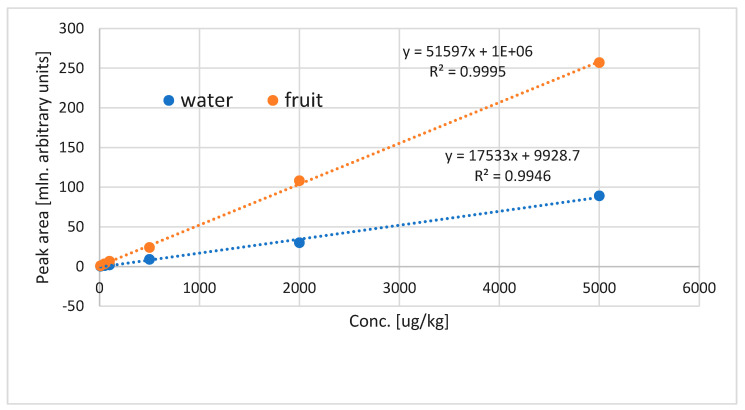
Influence of the matrix on calibration of ethyl butanoate: compounds added in a range of 10–5000 µg/kg, two matrices tested—water and blended fruit.

Extraction by VASE is used not only to monitor the profile of volatile compounds, but should also perform satisfactorily in quantitative analysis. It has been demonstrated previously to be a good quantitative method for volatile determination in beer and oil [1,2]. For quantitation in plant matrices, the key issue is the matrix influence on the extraction of volatiles, and matrix effects can usually be observed. For cape gooseberry, the existence of matrix effects was tested by preparing calibration curves in water and in blended fruit. For all compounds, there was a significant difference in the slopes of calibration curves, as can be seen in Figure 6. Clearly the matrix effects are to be considered in quantitative analysis via VASE.

For quantitation of the selected volatile compounds of interested detected by GC/MS in cape gooseberry, calibration curves were prepared by spiking appropriate concentrations of standards (dissolved in ethanol) into blended gooseberry to achieve concentrations of 10, 50, 100, 500, 2000, and 5000 µg/kg (fresh weight). Calibration curves were generated, with good linearity achieved within the tested concentration range for 22 compounds (Table 1). For the majority (19/22) of compounds, R^2^ > 0.99 values were noted. Interestingly, the linearity range was broader than the one tested for ITEX (from 5–25 µg/kg up to 500 µg/kg) [25]. This shows the excellent properties of VASE for quantification. The tested compounds varied in terms of their hydrophobicity, with LogP values ranging from 0.740 to 4.861, and they consisted of esters, terpenes, alcohols, and aldehydes. The reproducibility of the method was tested using five extractions via the same sorbent pen of a 100 µg/kg standard concentration spiked into the blended fruit.

As the standard compounds for the calibration curves were spiked into blended fruit, the quantitation could be based, in fact, on a standard addition method, as the calibration curves did not go through the origin. Based on this approach, the selected compounds were quantified and the results are shown in Table 1.

## 3. Materials and Methods

### 3.1. Analytical Equipment

Volatile compounds were extracted using a VASE 5800 system (Entech Instruments, Simi Valley, CA, USA). The idea and workflow of VASE extraction have been described before [2]. Sorbent pens used in the experiment were filled with Tenax TA (35/60). The VASE 5800 system was mounted on a GC/MS system (7890 A/7895 TAD MSD, Agilent Technologies, Santa Clara, CA, USA) equipped with DB-5MS column (30 m × 0.25 mm × 0.50µm, Agilent Technologies). Helium at the flow of 1 mL/min. was used as a carrier gas. The following GC oven program was used for analyses: initial temperature 40 °C for 3 min, then increase of 10 °C/min to 180 °C, and 20 °C/min. to 280 °C (kept for 4 min). The GC/MS transfer line was kept at 290 °C. The mass spectrometer was working in full-scan mode in a m/z range of 33–333, 6.6 scans/s. All peak area comparisons, as well as quantitation, were performed for TIC (total ion current) peaks. Mass Hunter version 07.00.0 was used to control the GC/MS system.

The following parameters were used for the VASE 5800 desorption system: Preheat; duration—2 min. at 260 °C, Injection; split (1:25) achieved by splitter frit, Desorption; standby temp. 70 °C, duration 15 min., desorption temperature 260 °C, desorption time 20 min., Bakeout; time 5 min. at 260 °C, Post bake; duration 4 min., temp. 70 °C.

### 3.2. Sample Preparation and VASE Extraction

Cape gooseberry fruit originating from Peru were purchased in a local grocery store. Blended neat fruit samples were weighed into 44 mL screw top amber vials and vials were capped with Teflon-lined caps. Before analysis, the Teflon-lined cap was replaced by a special Entech metal cap which could seal the sorbent pen for sampling. After inserting the sorbent pen into the vial, air was evacuated from the vial via the sorbent pen using a membrane pump with a manometer. Once the vacuum in the vial reached 28″ Hg (after approximately 15 s), the pump was disconnected and extraction of volatile compounds was performed; the sorbent pen was removed from the vial and manually transferred into the Entech injection port of GC/MS, where the desorption cycle started. The setup and all the steps were provided in [21] in its Supplementary files. To assess different extraction conditions, vials were heated in a dedicated sample heater (Entech Instruments, Simi Valley, CA, USA). Different sample sizes were tested (0.2 g, 0.5 g, 1 g, and 10 g), as well as two variants of extraction conditions: fast, 30 min extraction at 40 °C vs. long extraction (120 min.) at room temperature (20 °C). Evaluation of quantitative possibilities with VASE was based on blended fruit spiked with standard compounds detected by GC/MS in cape gooseberry fruit. Also, curves were prepared in water to look for the intensity of the matrix effect. Concentrations of particular compounds in a range of 10–5000 µg/kg were spiked into the matrix. To test the influence of Ag^+1^ ions on enzymatic reaction quenching, a solution of AgNO_3_ was added to the blended fruit as described in [31].

## 4. Conclusions

The presented experiments, facilitating VASE use for the extraction of *Physalis peruviana* L. volatiles, demonstrated that the technique provides a rich profile of compounds of different classes. A large, 10 g sample size was selected for analyses; however, no overloading of sorbent was observed and analyses were performed in split mode without any form of column preconcentration. The large capacity of the sorbent guarantees very good linearity in a broad (low µg/kg to several mg/kg) range. The profile, and overall the quantities of isolated compounds, are highly dependent on the extraction parameters, not only time/temperature, but also the tissue disintegration method. The method has potential for the isolation of volatiles from other fruit.

## Figures and Tables

**Table 1:** Compounds detected in *Physalis peruviana* L. and selected for quantitation using VASE.

Rt [min.]	Compound	LogP	Linearity [R^2^] *	RSD **	Amount [µg/kg]
3.057	2-methylpropanal	0.740	0.9994	14.8	117
7.258	Pentanal	1.423	0.9971	20.4	43
8.151	α-pinene	4.830	0.9438	3.4	748
8.700	Ethyl butanoate	1.804	0.9995	12.9	106
9.702	Hexanal	1.780	0.9980	15.8	130
10.071	β-pinene	4.366	0.9996	7.6	28
10.878	1-butanol	0.880	0.9713	4.6	1881
11.273	β-myrcene	4.170	0.9978	16.3	111
11.288	α-phellandrene	4.408	0.9767	6.3	326
11.969	Limonene	4.380	0.9994	7.4	55
11.998	2-methyl-1-butanol	1.280	0.9970	5.9	932
12.134	Eucalyptol	2.740	0.9987	14.9	45
12.569	Ethyl hexanoate	2.823	0.9910	16.5	149
12.841	Ocimene	4.700	0.9987	6.1	21
13.243	p-cymene	4.100	0.9972	11.7	58
13.430	α-terpinolene	3.280	0.9916	13.4	152
14.345	1-hexanol	2.030	0.9965	11.8	234
15.659	Ethyl octanoate	3.842	0.9964	15.1	5.8
17.005	Linalool	2.970	0.9929	8.4	134
17.094	Benzaldehyde	1.480	0.9995	6.8	61
17.966	Terpinene-4-ol	3.260	0.9998	10.4	232
18.264	Ethyl decanoate	4.861	0.9920	14.5	18

* Linearity was determined in a range of 10–5000 µg/kg (FW) for all compounds, ** RSD (relative standard deviation) was determined by five injections of 100 µg/kg of standard solution spiked into cape gooseberry blended matrix, (n = 5).

## Data Availability

Data are contained within the article.
